# Supplementary material for: Heat adaptation of phage T7 under an extended genetic code
Source: Virus Evol. 2021 Dec 1;7(2):veab100. doi: 10.1093/ve/veab100 (PMC8923235; doi:10.1093/ve/veab100)
Supplement: veab100_Supp [file veab100_supp.zip › Sequencing_Analysis.zip!/Sequencing_Analysis/S2/evidence/SNP_13.html]

BRESEQ :: Evidence


| Predicted mutation | | | | | | |
| --- | --- | --- | --- | --- | --- | --- |
| evidence | seq id | position | mutation | annotation | gene | description |
| RA | NC\_001604.1 | 34,975 | A→G | intergenic (–/–) | – / – | –/– |

| Read alignment evidence... | | | | | | | | | | | |
| --- | --- | --- | --- | --- | --- | --- | --- | --- | --- | --- | --- |
|  | seq id | position | | ref | new | freq | score (cons/poly) | reads | annotation | genes | product |
| \* | NC\_001604.1 | 34,975 | 0 | A | G | 98.8% | 15446.3 / NA | 5051 | intergenic (–/–) | *–/–* | –/– |
| Reads supporting (aligned to +/- strand):  **ref** base A (5/4);  **major** base G (2508/2460);  **minor** base T (48/14);  **total** (2573/2478) | | | | | | | | | | | |
| Fisher's exact test for biased strand distribution *p*-value = 1.80e-05 | | | | | | | | | | | |
| Kolmogorov-Smirnov test that lower quality scores support variant *p*-value = 1.00e+00 | | | | | | | | | | | |

Only 100 of 5051 total aligned reads displayed.  

`TGATTTTAATATCCCGTTTGAGTATCTAGCCCGTAAGTTCGTAGTGGTAACTCTTATTGGTGTAGACCGAAAGGTCCTTACGATTAATACAGACTATCGCTTTGCTACACGTACTACTATCTCTCTGACAAAGGCTTGGGGTCCAGCCGATGGCTACACGACCATCGAGTTACGTCGAGTAACCTCCACTACCGACCGATTGGTTGACTTTACGGATGGTTCAATCCTCCGCGCGTATGACCTTAACGTCGCTCAGATTCAAACGATGCACGTAGCGGAAGAGGCCCGTGACCTCACTACGGATACTATCGGTGTCAATAACGATGGTCACTTGGATGCTCGTGGTCGTCGAATTGTGAACCTAGCGAACGCCGTGGATGACCGCGATGCTGTTCCGTTTGGTCAACTAAAGACCATGAACCAGAACTCATGGCAAGCAC‑GTAATGAAGCCTTACAGTTCCGTAATGAGGCTGAGACTTTCAGAAACCAAGCGGAGGGCTTTAAGAACGAGTCCAGTACCAACGCTACGAACACAAAGCAGTGGCGCGATGAGACCAAGGGTTTCCGAGACGAAGCCAAGCGGTTCAAGAATACGG  >  NC_001604.1/34677‑35272`  

                                                                                                                                                                                                                                                                                                          `|`                                                                                                                                                                                                                                                                                                            
  `aTTTTCAAATCCCGTTGGAGTAGCTAGCCCGTAAGTTCGTAGTGGTAACTCTTATTGTTGTAGACCGAAAGGTCCTTACGATTAAGTCAAACTATCGCTTTGCTACACTTACTACTATCTCGCTTACAAAGGCTTGGGGTCCAGCCGATGGCTACACGACCATCGAGTTACGTCGAGTAACCTCCACTACCGACCGATTGGTTGACTTTACGGATGGTTCAATCCTCCGCGCGTATGACCTTAACGTCGCTCAGATTCAAACGATGCACGTAGCGGAAGAGGCCCGTGACCTCACTGCGGa                                                                                                                                                                                                                                                                                                        <  1:783040/301‑1 (MQ=255)`  
      `gagacagccGTTTGAGTATCTAGCCCGTAAGTTCGTAGTGGTAACTCTTATTGGTGTAGACCGAAAGGTCCTTACGATTAATACAGACTATCGCTTTGCTACACGTACTACTATCTCTCTGACAAAGGCTTGGGGTCCAGCCGATGGCTACACGACCATCGAGTTACGTCGAGTAACCTCCACTACCGACCGATTGGTTGACTTTACGGATGGTTCAATCCTCCGCGCGTATGACCTTAACGTCGCTCAGATTCAAACGATGCACGTAGCGGAAGAGGCCCGTGACCTCACTGCGGATACt                                                                                                                                                                                                                                                                                                    <  1:374104/294‑1 (MQ=255)`  
         `tatCCCGTTTGAGTATCTAGCCCGTAAGTTCGTAGTGGTAACTCTTATTGGTGTAGACCGAAAGGTCCTTACGATTAATACAGACTATCGATTTGCTACACGTACTACTATCTCTCTGACAAAGGCTTGGGGTCCAGCCGATGGCTACACGACCATCGAGTTACGTCGAGTAACCTCCACTACCGACCGATTGGTTGACTTTACGGATGGTTCAATCCTCCGCGCGTATGACCTTAACGTCGCTCAGATTCAAACGATGCACGTAGCGGAAGAGGCCCGTGACCTCACTGCGGATACTATc                                                                                                                                                                                                                                                                                                 <  1:276995/301‑1 (MQ=255)`  
           `tCCCGTTTGAGTATCTAGCCCGTAAGTTCGTAGTGGTAACTCTTATTGGTGTAGACCGAAAGGTCCTTACGATTAATACAGACTATCGCTTTGCTACACGTACTACTATCTCTCTGACAAAGGCTTGGGGTCCAGCCGATGGCTACACGACCATCGAGTTACGTCGAGTAACCTCCACTACCGACCGATTGGTTGACTTTACGGATGGTTCAATCCTCCGCGCGTATGACCTTAACGTCGCTCAGATTCAAACGATGAACGTAGCGGAAGAGGCCCGTGACCTCACTGCGGATACTATCgg                                                                                                                                                                                                                                                                                               <  1:210076/301‑1 (MQ=255)`  
           `tCCCGGTTGAGTATCTAGCCCGTAAGTTCGTAGTGGTAACTCTTATTGGTGTAGACCGAAAGGTCCTTACGATTAATACAGACTATCGCTTTGCTACACGTACTACTATCTCTCTGACAAAGGCTTGGGGTCCAGCCGATGGCTACACGACCATCGAGTTACGTCGAGTAACCTCCACTACCGACCGATTGGTTGACTTTACGGATGGTTCAATCCTCCGCGCGTATGACCTTAACGTCGCTCAGATTCAAACGATGCACGTAGCGGAAGAGGCCCGTGACCTCACTGCGGATACTATCgg                                                                                                                                                                                                                                                                                               <  1:244083/301‑1 (MQ=255)`  
               `gTTTGAGTATCTAGCCCGTAAGTTCGTAGTGGTAACTCTTATTGGTGTAGACCGAAAGGTCCTTACGATTAATACAGACTATCGCTTTGCTAAACGTACTACTATCTCTCTGACAAAGGCTTGGGGTCCAGCCGATGGCTACACGACCATCGAGTTACGTCGAGTAACCTCCACTACCGACCGATTGGTTGACTTTACGAATGGTTCAATCCTCCGCGCGTATGACCTTAACGTCGCTCAGATTCAAACGATGCACGTAGCGGAAGTGGCACGTGACCTCACTGCGGATGCTATCGGTGTc                                                                                                                                                                                                                                                                                           >  1:672967/1‑301 (MQ=255)`  
                `tttGAGTATCTAGCCCGTAAGTTCGTAGTGGTAACTCTTATTGGTGTAGACCGAAAGGTCCTTACGATTAATACAGACTATCGCTTTGCTACACGTACTACTATCTCTCTGACAAAGGCTTGGGGTCCAGCCGATGGCTACACGACCATCGAGTTACGTCGAGTAACCTCCACTACCGACCGATTGGTTGACTTTACGGATGGTTCAATCCTCCGCGCGTATGACCTTAACGTCGCTCAGATTCAAACGATGCACGTAGCGGAAGAGGCCCGTGACCTCCCTGCGGATACTATCGGTGTCa                                                                                                                                                                                                                                                                                          >  1:485076/1‑301 (MQ=255)`  
                     `gTATCTAGCCCGTAAGTTCGTAGTGGTAACTCTTATTGGTGTAGACCGAAAGGTCCTTACGATTAATACAGACTATCGCTTTGCTACACGTACTACTATCTCTCTGACAAAGGCTTGGGGTCCAGCCGATGGCTACACGACCATCGAGTTACGTCGAGTAACCTCCACTACCGACCGATTGGTTGACTTTACGGATGGTTCAATCCTCCGCGCGTATGACCTTAACGTCGCTCAGATTCAAACGATGCACGTAGCGGAAGAGGCCCGTGACCTCGCTGCGGATACTATCGGTGTCAATAAc                                                                                                                                                                                                                                                                                     <  1:379964/301‑1 (MQ=255)`  
                                   `aGTTCGTAGTGGTAACTCTTATTGGTGTAGACCGATAGGTCCTTACGATTAATACAGACTATCGATTTGCTACACGTACTACTATCCCTCTGACAAAGGCTTGGGGTCCAGCCGATGGCTACACGACTATCGAGTTATGTCGAGTAAACTCCACTACAGACCGATTGGTTGACTTAACGGATGGTTCAATCCTCCGCGCGTATGACTTTAACGACTCTCAGATTCAAAAGATGCCTGTAGCGGAAGAGGCCCGTGACCTCACTGCGGATAATATCGGTGTCAATAACGACGGGCACTAGGa                                                                                                                                                                                                                                                                       >  1:27021/1‑301 (MQ=255)`  
                                             `ggTAACTCTTATTGGTGTAGACCGAAAGGTCCTTACGATTAATACAGACTATCGCTTTGCTACACGTACTACTATCTCTCTGACAAAGGCTTGGGGTCCAGCCGATGGCTACACGACCATCGAGTTACGTCGAGTAACCTCCACTACCGACCGATTGGTTGACTTTACGGATGGTTCAATCCTCCGCGCGTATGACCTTAACGTCGCTCAGATTCAAACGATGCACGTAGCGGAAGAGGCCCGTGACATCACTGCGGATACTATCGGTGTCAATAACGATGGTCACTTGGATGCTCGTGgt                                                                                                                                                                                                                                                             >  1:395701/1‑301 (MQ=255)`  
                                                  `ctctTATTTGTGTAGACCGAAAGGTCCTTACGATTAATACAGACTATCGCTTTGCTACACGTACTACTATCTCTCTGACAAAGGCTTGGGGTCCAGCCGATGGCTACACGACCATCGAGTTACGTCGAGTAACCTCCACTACCGACCGATTGGTTGACTTTACGGATGGTTCAATCCTCCGCGCGTATGACCTTAACGTCGCTCAGATTCAAACGATGCACGTAGCGGAAGATGCCCGTGACCTCACTTCGGATACTATCGGTGTCAATAACGATTGTCACTTGGATGCCCGTGGtcgtcg                                                                                                                                                                                                                                                        >  1:759399/1‑301 (MQ=255)`  
                                                    `ctTATTGGTGTAGACCGAAAGGTCCTTACGATTAATACAGACTATCGCTTTGCTACACGTACTACTATCTCTCTGACAAAGGCTTGGGGTCCAGCCGATGGCTACACGACCATCGAGTTACGTCGAGTAACCTCCACTACCGACCGATTGGTTGACTTTACGGATGGTTCAATCCTCCGCGCGTATGACCTTAACGTCGCTCAGATTCAAACGATGCACGTAGCGGAAGAGGCCCGTGACCTCACTGCGGATACTATCGGTGTCAATAACGATGGTCACTTGGATGCTCGTGGTCGTCGaa                                                                                                                                                                                                                                                      >  1:122417/1‑301 (MQ=255)`  
                                                    `ctTATTGGTGTAGACCGAAAGGTCCTTACGATTAATACAGACTATCGCTTTGCTACACGTACTACTATCTCTCTGACAAAGGCTTGGGGTCCAGCCGATGGCTACACGACCATCGAGTTACGTCGAGTAACCTCCACTACCGACCGATTGGTTGACTTTACGGATGGTTCAATCCTCCGCGCGTATGACCTTAACGTCGCTCAGATTCAAACGATGCACGTAGCGGAAGAGGCCCGTGACCTCACTGCGGATACTATCGGTGTCAATAACGATGGTCACTTGGATGCTCGTGGTCGTCGaa                                                                                                                                                                                                                                                      >  1:513938/1‑301 (MQ=255)`  
                                                      `tATTGGTGTAGACCGAAAGGTCCTTACGATTAATACAGACTATCGCTTTGCTACACGTACTACTATCTCTCTGACAAAGGCTTGGGGTCCAGCCGATGGCTACACGACCATCGAGTTACGTCGAGTAACCTCCATTACCGACCGATTGGTTGACTTTACGGATGGTTCAATCCTCCGCGCGTATGACCTTAACGTCGCTCAGATTCAAACGATGCACGTAGCGGAAGAGGCCCGTGACCTCACTGCGGATACTATCGGTGTCAATAACGATGGTCCCTTGGATGCCCGTGGTCGTCGAAtt                                                                                                                                                                                                                                                    >  1:563811/1‑301 (MQ=255)`  
                                                          `ggTGTAGACCGAAAGGTCCTTACGATTAATACAGACTATCGCTTTGCTACACGTACTACTATCTCTCTGACAAAGGCTTGGGGTCCAGCCGATGGCTACACGACCATCGAGTTACGTCGAGTAACCTCCACTACCGACCGATTGGTTGACTTTACGGATGGTTCAATCCTCCGCGCGTATGACCTTAACGTCGCTCAGATTCAAACGATGCACGTAGTGGAAGAGGCCCGTGACCTCACTGCGGATACTATCGTTGTCAATAACGATGGTCACTTGGATGCTCGTGGTCGTCGAATTGTGa                                                                                                                                                                                                                                                >  1:367398/1‑301 (MQ=255)`  
                                                          `ggTGTAGACCGAAAGGTCCTTACGATTAATACAGACTATCGCTTTGCTACACGTACTACTATCTCTCTGACAAAGGCTTGGGGTCCAGCCGATGGCTACACGACCATCGAGTTACGTCGAGTAACCTCCACTACCGACCGATTGGTTGACTTTACGGATGGTTCAATCCTCCGCGCGTATGACCTTAACGTCGCTCAGATTCAAACGATGCACGTAGCGGAAGAGGCCCGTGACCTCACTGCGGATACTATCGGTGTCAATAACGATGGTCACTTGGATGCTCGTGGTCGTCGAATTGTGa                                                                                                                                                                                                                                                >  1:157257/1‑301 (MQ=255)`  
                                                          `ggTGTAGACCGAAAGGTCCTTACGATTAATACAGACTATCGCTTTGCTACACGTACTACTATCTCTCTGACAAAGGCTTGGGGTCCAGCCGATGGCTACACCACCATCGAGTTACGTCTAGTAACCTCCACTACCGACCGATTGTTTTACTTTACGGATGGTTCAATCCTCCGCGCGTATGACCTTAACCTCGCCCAGATTCAAACGATGCACGTAGCGGAAGAGGCCCGTTACCTCACTGCGGATACTATCGGTGTCAATAACGATGGTCACTTTGATGCTCGTGGTCGTCGAATtgctt                                                                                                                                                                                                                                                >  1:283427/1‑298 (MQ=255)`  
                                                           `gtgtAGACCGAAAGGTCCTTACGATTAATACAGACTATCGCTTTGCTACACGTACTACTATCTCTCTGACAAAGGCTTGGGGTCCAGCCGATGGCTACACGACCATCGAGTTACGTCGAGTAACCTCCACTACCGACCGATTGGTTGACTTTACGGATGGTTCAATCCTCCGCGCGTATGACCTTAACGTCGCTCAGATTCAAACGATGCACGTAGCGGAAGAGGCCCGTGACCTCACTGCGGATACTATCGGTGTCAATAACGATGGTCACTTGGATGCTCGTGGTCGTCGAATTGTGaa                                                                                                                                                                                                                                               >  1:652646/1‑301 (MQ=255)`  
                                                           `gtgtAGACCGAAAGGTCATTACGATTAATACAGACTATCGCTTTGCTACACGTACTACTATCTCTCTGACAAAGGCTTGGGGTCCAGCCGATGGCTACACGACCATCGAGTTACGTCGAGTAACCTCCACTACCGACCGATTGGTTGACTTTACGGATGGTTCAATCCTCCGCGCGTATGACCTTAACGTCGCTCAGATTCAAACGATGCACGTAGCGGAAGAGGCCCGTGACCTCACTGCGGATACTATCGGTGTCAATAACGATGGTCACTTGGATGCTCGTGGTCGTCGAATTGTGaa                                                                                                                                                                                                                                               >  1:556084/1‑301 (MQ=255)`  
                                                            `tgtAGACCGAAAGGTCCTTACGATTAATACAGACTATCGCTTTGCTACACGTACTACTATCTCTCTGACAAAGGCTTGGGGTCCAGCCGATGGCTACACGACCATCGAGTTACGTCGAGTAACCTCCACTACCGACCGATTGGTTGACTTTACGGATGGTTCAATCCTCCGCGCGTATGACCTTAACGTCGCTCAGATTCAAACGATGCACGTAGCGGAAGAGGCCCGTGACCTCACTGCGGATACTATCGGTGTCAATAACGATGGTCACTTGGATGCTCGTGGTCGTCGAATTGTGAAc                                                                                                                                                                                                                                              <  1:222667/301‑1 (MQ=255)`  
                                                             `gtAGACCGAAAGGTCCTTACGATTAATACAGACTATCGCTTTGCTACACGTACTACTATCTCTCTGACAAAGGCTTGGGGTCCAGCCGATGGCTACACGACCATCGAGTTACGTCGAGTAACCTCCACTACCGACCGATTGGTTGACTTTACGGATGGTTCAATCCACCGCGCGTATGACCTTAACGTCGCTCAGATTCAAACGATGCACGTAGCGGAAGAGGCCCGTGACCTCACTGCGGATACTATCGGTGTCAATAACGATGGTCACTTGGATGCTCGTGGTCGTCGAATTGTGAAcc                                                                                                                                                                                                                                             >  1:689615/1‑301 (MQ=255)`  
                                                                `gACCGAAAGGTCCTTACGATTAATACAGACTATCGCTTTGCTACACGTACTACTATCTCTCTGACAAAGGCTTGGGGTCCAGCCGATGGCTACACGACCATCGAGTTACGTCGAGTAACCTCCACTACCGACCGATTGGTTGACTTTACGGATGGTTCAATCCTCCGCGCGTATGACCTTAACGTCGCTCAGATTCAAACGATGCACGTAGCGGAAGAGGCCAGTGACCTCACTGCGGATAATATCGGTGCCAATAACGATGGTCACTTGGATGCTCGTGGTCGTCGAATTTTGAACCTAg                                                                                                                                                                                                                                          >  1:477864/1‑301 (MQ=255)`  
                                                                            `cTTACGATTAATACAGACTATCGCTTTGCTACACGTACTACTATCTCTCTGACAAAGGCTTGGGGTCCAGCCGATGGCTACACGACCATCGAGTTACGTCGAGTAACCTCCACTACCGACCGATTGGTTGACTTTACGGATGGTTCAATCCTCCGCGCGTATGACCTTAACGTCGCTCAGATTCAAACGATGCACGTAGCGGAAGAGGCCCGTGACCTCACTGCGGATACTATCGGTGTCAATAACGATGGTCACTTGGATGCTCGTGGTCGTCGAATTGTTAACCTAGCGAACGCCGttg                                                                                                                                                                                                                              >  1:528676/1‑299 (MQ=255)`  
                                                                             `ttACGATTAATACAGACTATCGCTTTGCTACACGTACTACTATCTCTCTGACAAAGGCTTGGGGTCCAGCCGATGGCTACACGACCATCGAGTTACGTCGAGTAACCTCCACTACCGACCGATTGGTTGACTTTACGGATGGTTCAATCCTCCGCGCGTATGACCTTAACGTCGCTCAGATTCAAACGATGCACGTAGCGGAAGAGGCCCGTGACCTAACTGCGGATACTATCGGTGTCAATAACGATGGTCACTTGGATGCTCGTGGTCGTCGAATTGTGAACCTAGCGAACGCCGTGGa                                                                                                                                                                                                                             <  1:257627/301‑1 (MQ=255)`  
                                                                             `ttACGATTAATACAGACTATCGCTTTGCTACACGTACTACTATCTCTCTGACAAAGGCTTGGGGTCCAGCCGATGGCTACACGACCATCGAGTTACGTCGAGTAACCTCCACTACCGACCGATTGGTTGACTTTACGGATGGTTCAATCCTCCGAGCGTATGACCTTAACGTCGCTCAGATTCAAACGATGCACGTAGCGGAAGAGGCCCGTGACCTCACTGCGGATACTATCGGTGTCAATAACGATGGTCACTTGGATGCTCGTGGTCGTCGAATTGTGAACCTAGCGAACGCCGTGGa                                                                                                                                                                                                                             <  1:179158/301‑1 (MQ=255)`  
                                                                               `agatgtgtataagagacaggccttGCTACACGTACTACTATCTCTCTGACAAAGGCTTGGGGTCCAGCCGATGGCTACACGACCATCGAGTTACGTCGAATAACCTCCACTACCGACCGATTGGTTGACTTTACGGATGGTTCAATCCTCCGCGCGTATGACCTTAACGTCGCTCAGATTCAAACGATGCACGTAGCGGAAGAGGCCCGTGACCTCACTGCGGATACTATCGGTGTCAATAACGATGGTCACTTGGATGCTCGTGGTCGTCGAATTGTGAACCTAGCGAACGCCGTGGATg                                                                                                                                                                                                                           <  1:796695/279‑1 (MQ=255)`  
                                                                                 `gATTAATACAGACTATCGCTTTGCTACACGTACTACTATCTCTCTGACAAAGGCTTGGGGTCCAGCCGATGGCTACACGACCATCGAGTTACGTCGAGTAACCTCCACTACCGACCGATTGGTTGACTTTACGGATGGTTCAATCCTCCGCGCGTATGACCTTAACGTCGCTCAGATTCAAACGATGCACGTAGCGGAAGAGGCCCGTGACCTCACTGCGAATACTATCGGTGTCAATAACGATGGTCACTTGGATGCTCGTGGTCGTCGAATTGTGAACCTAGCGAACGCCGTGGATgct                                                                                                                                                                                                                         >  1:402343/1‑299 (MQ=255)`  
                                                                                   `agagacaggACTATCGCTTTGCTACACGTACTACTATCTCTCTGACAAAGGCTTGGGGTCCAGCCGATGGCTACACGACCATCGAGTTACTTCGAGTAACCTCCACTACCGACCGATTGGTTGACTTTACGGATGGTTCAATCCTCCGCGCGTATGACCTTAACGTCGCTCAGATTCAAACGATGCACGTAGCGGAAGAGGCCCGTGACCTCACTGCGGATACTATCGGTGTCAATAACGATGGTCACTTGGATGCTCGTGGTCGTCGAATTGTGAACCTAGCGAACGCCGTGGATGACcg                                                                                                                                                                                                                       <  1:85357/293‑1 (MQ=255)`  
                                                                                     `aaTACAGACTATCGCTTTGCTACACGTACTACTATCTCTCTGACAAAGGCTTGGGGTCCAGCCGATGGCTACACGACCATCGAGTTACGTCGAGTAACCTCCACTACCGACCGATTGGTTGACTTTACGGATGGTTCAATCCTCCGCGCGTATGACCTTAACGTCGCTCAGATTCAAACGATGCACGTAGCGGAAGAGGCCCGTGACCTCACTGCGGATACTATCGGTGTCAATAACGATGGTCACTTGGATGCTCGTGGTCGTCGAATTGTGAACCTAGCGAACGCCGTGGactgtctct                                                                                                                                                                                                                     >  1:41014/1‑293 (MQ=255)`  
                                                                                      `aTACAGACTATCGCTTTGCTACACGTACTACTATCTCTCTGACAAAGGCTTGGGGTCCAGCCGATGGCTACACGACCATCGAGTTACGTCGAGTAACCTCCACTACCGACCGATTGGTTGACTTTACGGATGGTTAAATCCTCCGCGCGTATGACCTTAACGTCGCTCAGATTCAAACGATGCACGTAGCGGAAGAGGCCCGTGACCTCACTGCGGATAATATCGGTGTCAATAACGATGGTCACTTGGATGCTCGTGGTCGTCGAATTGTGAACCTAGCGAACGCCGTGGATGACCGCGa                                                                                                                                                                                                                    <  1:10016/301‑1 (MQ=255)`  
                                                                                        `gagacagaTCGCTTTGCTACACGTACTACTATCTCTCGGACAAAGGCTTGGGGTCCAGCCGATGGCTACACGACCATCGAGTTACGTCGAGTAACCTCCACTACCGACCGATTGGTTGACTTTACGGATGGTTCAATCCTCCGCGCGTATGACCTTAACGTCGCTCAGATTCAAACGATGCACGTAGCGGAAGAGGCCCGTGACCTCACTGCGGATACTATCGGTGTCAATAACGATGGTCACTTGGATGCTCGTGGTCGTCGAATTGTGAACCTAGCGAACGCCGTGGATGACCGCGATg                                                                                                                                                                                                                  <  1:594550/294‑1 (MQ=255)`  
                                                                                         `cttggagatgtgtataagagacagctactaACTCTCTGACAAAGGCTTGGGGTCCAGCCGATGGCTACACGACCATCGAGTTACGTCGAGTAACCTCCACTACCGGCCGATTGGTTGACTTTACGGATGGTTCAATCCTCCGCGCGTATGACCTTAACGTCGCTCAGATTCAAACGATGCACGTAGCGGAAGAGGCCCGTGACCTCACTGCGGATACTATCGGTGTCAATAACGATGGTCACTTGGATGCTCGTGGTCGTCGAATTGTGAACCTAGCGAACGCCGTGGATGACCGCGATGc                                                                                                                                                                                                                 <  1:116245/277‑1 (MQ=255)`  
                                                                                         `cAGACTATCGCTTTGCTACACGTACTACTATCTCTCTGACAAAGGCTTGGGGTCCAGCCGATGGCTACACGACCATCGAGTTACGTCGAGTAACCTCCACTACCGACCGATTGGTTGACTTTACGGATGGTTCAATCCTCCGCGCGTATGACCTTAACGTCGCTCAGATTCAAACGATGCACGTAGCGGAAGAGGCCCGTGACCTCACTGCGGATACTATCGGTGTCAATAACGATGGTCACTTGGATGCTCGTGGTCGTCGAATTGTGAACCTAGCGAACGCCGTGGATGACCGCGATGc                                                                                                                                                                                                                 <  1:571708/301‑1 (MQ=255)`  
                                                                                         `cAGACTATCGCTTTGCTACACGTACTACTATCTCTCTGACAAAGGCTTGGGGTCCAGCCGATGGCTACACGACCATCGAGTTACGTCGAGTAACCTCCACTACCGACCGATTGGTTGACTTTACGGATGGTTCAATCCTCCGCGCGTATGACCTTAACGTCGCTCAGATTCAAACGATGCACGTAGCGGAAGAGGCCCGTGACCTCACTGCGGATACTATCGGTGTCAATAACGATGGTCACTTGGATGCTCGTGGTCGTCGAATTGTGAACCTAGCGAACGCCGTGGATGACCGCGATGc                                                                                                                                                                                                                 <  1:681703/301‑1 (MQ=255)`  
                                                                                           `gACTATCGCTTTGCTACACGTACTACTATCTCTCTGACAAAGGCTTGGGGTCCAGCCGATGGCTACACGACCATCGAGTTACGTCGAGTAACCTCCACTACCGACCGATTGGTTGACTTTACGGATGGTTCAATCCTCCGCGCGTATGACCTTAACGTCGCTCAGATTCAAACGATGCACGTAGCGGAAGAGGCCCGTGACCTCCCTGCGGCTACTATCGGTGTCAATAACGATGGTCACTTGGATGCTCGTGGTCGTCGAATTGTGAACCTAGCGAACGCCGTGGGTGAcctgtctcttt                                                                                                                                                                                                               >  1:23051/1‑292 (MQ=255)`  
                                                                                              `aagagacaggCTACAAGTACTACTATCTCTCTGACAAATGCTTGGGGTCCAGCCGATGGCTACACGACCATCGAGTTACGTCGAGTAACCTCCACTACCGACCGATTGGTTGACTTTACGGATGGTTCAATCCTCCGCGCGTATGACCTTAACGTCGCTCAGATTCAAACGATGAACGTAGCGGAAGAGGCCCGTGACCTCACTGCGGATACTATCGGTGTCAATAACGATGGTCACTTGGATGCTCGTGGTCGTCGAATTGTGAACCTAGCGAACGCCGTGGATGACCGCGATGCTGTTc                                                                                                                                                                                                            <  1:534770/292‑1 (MQ=255)`  
                                                                                               `aTCGCTTTGCTACACGTACTACTATCTCTCTGACAAAGGCTTGGAGTCCAGCCGATGGCTACACGACCATCGAGTTACGTCGAGTAACCTCCACTACCGACCGATTGGTTGACTTTACGGATGGTTCAATCCTCCGCACGTATGACCTTAACGTCGCTCAGATTCAAACGATGCACGTAGCGGAAGAGGCCCGTGACCTCACTGCGGATACTATCGGTGTCAATAACGATGGTCATTTGGATGCTCGTGGTCGTCGAATTGTGAACCTAGCGAACGCCGTGGATGACCGCGATGCTGTTcc                                                                                                                                                                                                           >  1:342439/1‑301 (MQ=255)`  
                                                                                                 `cGCTTTGCTACACGTACTACTATCTCTCTGACAAAGGCTTGGGGTCCAGCCGATGGCTACACGACCATCGAGTTACGTCGAGTAACCTCCACTACCGACCGATTGGTTGACTTTACGGATGGTTCAATCCTCCGCGCGTATGACCTTAACGTCGCTCAGATTCAAACGATGCACGTAGCGGAAGAGGCCCGTGACCTCACTGCGGATACTATCGGTGTCAATAACGATGGTCACTTGGATGCTCGTGGTCGTCGAATTGTGAACCTAGCGAACGCCGTGGATGACCGCGATGCTGTTcctg                                                                                                                                                                                                         >  1:41696/1‑299 (MQ=255)`  
                                                                                                   `cTTTGCTACACGTACTACTATCTCTCTGACAAAGGCTTGGGGTCCAGCCGATGGCTACACGACCATCGAGTTACGTCGAGTAACCTCCACTACCGACCGATTGGTTGACTTTACGGATGGTTCAATCCTCCGCGCGTATGACCTTAACGTCGCTCAGATTCAAACGATGCACGTAGCGGAAGAGGCCCGTGACCTCACTGCGGATACTATCGGTGTCAATAACGATGGTCACTTGGATGCTCGTGGTCGTCGAATTGTGAACCTAGCGAACGACGTGGATGACCGCGATGCTGTTCCGttt                                                                                                                                                                                                       <  1:171134/301‑1 (MQ=255)`  
                                                                                                     `ttGCTACACGTACTACTATCTCTCTGACAAAGGCTTGGGGTCCAGCCGATGGCTACACGATCATCGAGTTACGTCGAGTAACCTCCACTACCGACCAATTGGTTGACTTTACGGATGGTTCAATCCTCCGCGCGTATGACCTTAACGTCGCTCAGATTCAAACGATGCACGTAGCGGAAGAGGCCCGTGACCTCACTGCGGATACTATCGGTGTCAATAACGATGGTCACTTGGATGCTCGTGGTCGTCGAATTGTGAACCTAGCGAACGCCGTGGATGACCGCGATGCTGTTcctgtctc                                                                                                                                                                                                     >  1:187145/1‑295 (MQ=255)`  
                                                                                                     `ttGCTACACGTACTACTATCTCTCTGACAAAGGCTTGGGGTCCAGCCGATGGCTACACGACCATCGAGTTACGTCGAGTAACCTCCACTACCGACCGATTGGTTGACTTTACGGATGGTTCAATCCTCCGCGCGTATGACCTTAACGTCGCTCAGATTCAAACGATGCACGTAGCGGAAGAGGCCCGTGACCTCACTGCGGATACTATCGGTGTCAATAACGATGGTCACTTGGATGCTCGTGGTCGTCGAATTGTGAACCTAGCGAACGCCGTGGATGACCGCGATGCTGTTCCGTTTgg                                                                                                                                                                                                     >  1:76807/1‑301 (MQ=255)`  
                                                                                                     `ttGCTACACGTACTACTATCACTCTGACAAAGGCTTGGGGGCCAGTCGATGGCTACACGACCATCGAGTTACGGCAAGTAACCTACACTACCGACCGATTGGTTGACTTTACGGATGGTTCAATCCTCCGCGCGTATGACCTTAACGTCGCTCAGATTCAAACGATGCACGTAGCGAAAGAGGCCCGTGACCTCACTGCGGATACTATCGGTGTCAATAACGATGGTCACTTGGATGCTCGTGGTCGTCGAATTGTGAACCTAGCGAACGCCATGGATGACCGCGATGCTGTTCCGTGTgg                                                                                                                                                                                                     <  1:217160/301‑1 (MQ=255)`  
                                                                                                          `acacGTACTACTATCTCTCTGACAAAGGCTTGGGGTCCAGCCGATGGCTACACGACCATCGAGTTACGTCGAGTAACCTCCACTACCGACCGATTGGTTGACTTTACGGATGGTTCAATCCTCCGCGCGTATGACCTTAACGTCGCTCAGATTCAAACGATGCACGTAGCGGAAGAGGCCCGTGACCTCACTGCGGATACTATCGGTGTCAATAACGATGGTCACTTGGATGCTCGTGGTCGTCGAATTGTGAACCTAGCGAACGCCGTGGATGACCGCGATGCTGTTCCGTTTGGTCAAc                                                                                                                                                                                                <  1:750866/301‑1 (MQ=255)`  
                                                                                                           `cacGTACTACTATCTCTCTGACAAAGGCTTGGGGTCCAGCCGATGGCTACACGACCATCGAGTTACGTCGAGTAACCTCCACTACCGACCGATTGGTTGACTTTACGGATGGTTCAATCCTCCGCGCGTATGACCTTAACGTCGCTCAGATTCAAACGATGCACGTAGCGGAAGAGGCCCGTGACCTCACTGCGGATACTATCGGTGTCAATAACGATGGTCACTTGGATGCTCGTGGTCGTCGAATTGTGAACCTAGCGAACGCCGTGGATGACCGCGATGCTGTTCCGTTTGGTCAACt                                                                                                                                                                                               >  1:587626/1‑301 (MQ=255)`  
                                                                                                           `agcGTACTACTATCTCTCTGACAAAGGCTTGGGGTCCAGTCGATGGCTACACGACCATCGAGTTACGTCGAGTAACCTCCACTACCGACCGATTGGTTGACTTTACGGATGGTTCAATCCTCCGCGCGTATGACCTTAACGTCGCTCAGATTCAAACGATGCACGTAGCGGAAGAGGACCGTGACCTCACTGCGGATACTATCGGTGTCAATAACGATGGTCACTTGGATGCTCGTGGTCGTCGAATTGTGAACCTAGCGAACGCCGTGGATGACCGCGATGCTGTTCCGTTTGGTCAACt                                                                                                                                                                                               <  1:350136/299‑1 (MQ=255)`  
                                                                                                                 `gagacagctctctGACAAAGGCTTGGGGTCCAGCCGATGGCTACACGACCATCGAGTTACGTCGAGTAACCTCCACTACCGACCGATTGGTTGACTTTACGGATGGTTCAATCCTCCGCGCGTATGACCTTAACGTCGCTCAGATTCAAACGATGCACGTAGCGGAAGAGGCCCGTGACCTCACTGCGGATACTATCGGTGTCAATAACGATGGTCACTTGGATGCTCGTGGTCGTCGAATTGTGAACCTAGCGAACGCCGTGGATGACCGCGATGCTGTTCCGTTTGGTCAACTAAAGAc                                                                                                                                                                                         <  1:5004/294‑1 (MQ=255)`  
                                                                                                                 `ctactaTCTCTCTGACAAAGGCTTGGGGTCCAGCCGATGGCTACACGACCATCGAGTTACGTCGAGTAACCTCCACTACCGACCGATTGGTTGACTTTACGGATGGTTCAATCCTCCGCGCGTATGACCTTAACGTCGCTCAGATTCAAACGATGCACGTAGCGGAAGAGGCCCGTGACCTCACTGCGGATACTATCGGTGTCAATAACGATGGTCACTTGGATGCTCGTGGTCGTCGAATTGTGAACCTAGCGAACGCCGTGGATGACCGCGATGCTGTTcctggctcttatacacatct                                                                                                                                                                                         >  1:710395/1‑283 (MQ=255)`  
                                                                                                                 `ctactaTCTCTCTGACAAAGGCTTGGGGTCCAGCCGATGGCTACACGACCATCGAGTTACGTCGAGTAACCTCCACTACCGACCGATTGGTTGACTTTACGGATGGTTCAATCCTCCGCGCGTATGACCTTAACGTCGCTCAGATTCAAACGATGCACGTAGCGGAAGAGGCCCGTGACCTCACTGCGGATACTATCGGTGTCAATAACGATGGTCACTTGGATGCTCGTGGTCGTCGAATTGTGAACCTAGCGAACGCCGTGGATGACCGCGATGCTGTTCCGTTTGGTCAACTAAAGAc                                                                                                                                                                                         <  1:194579/301‑1 (MQ=255)`  
                                                                                                                 `ctactaTCTCTCTGACAAAGGCTTGGGGTCCAGCCGATGGCTACACGACCATCGAGTTACGTCGAGTAACCTCCACTACCGACCGATTGGTTGACTTTACGGATGGTTCAATCCTCCGCGCGTATGACCTTAACGTCGCTCAGATTCAAACGATGCACGTAGCGGAAGAGGCCCGTGACCTCACTGCGGATACTATCGGTGTCAATAACGATGATCACTTGGATGCTCGTGGTCGTCGAATTGTGAACCTAGCGAACGCCGTGGATGACCGCGATGCTGTTCCGTTTGGTCAACTAAAGAc                                                                                                                                                                                         >  1:667235/1‑301 (MQ=255)`  
                                                                                                                  `tactaTCTCTCTGACAAAGGCTTGGGGTCCAGCCGATGGCTACACGACCATCGAGTTACGTCGAGTAACCTCCACTACCGACCGATTGGTTGACTTTACGGATGGTTCAATCCTCCGCGCGTATGACCTTAACGTCGCTCAGATTCAAACGATGCACGTAGCGGAAGAGGCCCGTGACCTCACTGCGGATACTATCGGTGTCAATAACGATGGTCACTTGGATGCTCGTGGTCGTCGAATTGTGAACCTAGCGAACGCCGTGGATGACCGCGATGCTGTTCCGTTTGGTCAACTAAAGAcc                                                                                                                                                                                        <  1:78998/301‑1 (MQ=255)`  
                                                                                                                      `aTCTCTCTGACAAAGGCTTGGGGTCCAGCCGATGGCTACACGACCATCGAGTTACGTCGAGTAACCTCCACTACCGACCGATTGGTTGACTTTACGGATGGTTCAATCCTCCGCGCGTATGACCTTAACGTCGCTCAGATTCAAACGATGCACGTAGCGGAAGAGGCCCGTGACCTCACTGCGGATACTATCGGTGTCAATAACGATGGTCACTTGGATGCTCGTGGTCGTCGAATTGTGAACCTAGCGAACGCCGTGGATGACCGCGATGCTGTTCCGTTTGGTCAACTAAAGACCATgg                                                                                                                                                                                    >  1:150913/1‑300 (MQ=255)`  
                                                                                                                      `aTCTCTCTGACAAAGGCTTGGGGTCCAGCCGATGGCTACACGACCATCGAGTTACGTCGAGTAACCTCCACTACCGACCGATTGGTTGACTTTACGGATGGTTCAATCCTCCGCGCGTATGACCTTAACGTCGCTCAGATTCAAACGATGCACGTAGCGGAAGAGGCCCGTGACCTCACTGCGGATACTATCGGTGTCAATAACGATGGTCACTTGGATGCTCGTGGTCGTCGAATTGTGAACCTAGCGAACGCCGTGGATGACCGCGATGCTGTTCCGTTTGGTCAACTAAAGACCATgg                                                                                                                                                                                    >  1:358152/1‑300 (MQ=255)`  
                                                                                                                         `tctctGACAAAGGCTTGGGGTCCAGCCGATGGCTACACGACCATCGAGTTACGTCGAGTAACCTCCACTACCGACCGATTGGTTGACTTTACGGATGGTTCAATCCTCCGCGCGTATGACCTTAACGTCGCTCAGATTCAAACGATGCACGTAGCGGAAGAGGCCCGTGACCTCACTGCGGATACTATCGGTGTCAATAACGATGGTCACTTGGATGCTCGTGGTCGTCGAATTGTGAACCTAGCGAACGCCGTGGATGACCGCGATGCTGTTCCGTTTGGTCAACTAAAGACCATGGAcc                                                                                                                                                                                 <  1:92664/301‑1 (MQ=255)`  
                                                                                                                              `gACAAAGGCTTGGGGTCCAGCCGATGGCTACACGACCATCGAGTTACGTCGAGTAACCTCCACTACCGACCGATTGGTTGACTTTACGGATGGTTCAATCCTCCGCGCGTATGACCTTAACGTCGCTCAGATTCAAACGATGCACGTAGCGGAAGAGGCCCGTGACCTCACTGCGGATACTATCGGTGTCAATAACGATGGTCACTTGGATGCTCGTTGTCGTCGAATTGTGAACCTAGCGAACGCCGTGGATGACCGCGATGCTGTTCCGTTTGGTCAACTAAAGACCATGGACCAGAAc                                                                                                                                                                            <  1:424894/301‑1 (MQ=255)`  
                                                                                                                               `aCAAAGGCTTGGGGTCCAGCCGATGGCTACACGACCATCGAGTTACGTCGAGTAACCTCCACTACCGACCGATTGGTTGACTTTACGGATGGTTCAATCCTCCGCGCGTATGACCTTAACGTCGCTCAGATTCAAACGATGCACGTAGCGGAAGAGGCCCGTGACCTCACTGCGGATACTATCGGTGTCAATAACGATGGTCACTTGGATGCTCGTGGTCGTCGAATTGTGAACCTAGCGAACGCCGTGGATGACCGCGATGCTGTTCCGTTTGGTCAACTAAAGACCATGGACCAGAACt                                                                                                                                                                           <  1:602873/301‑1 (MQ=255)`  
                                                                                                                                           `ggTCCAGCCGATGGCTACACGACCATCGAGTTACGTCGAGTAACCTCCACTACCGACCGATTGGTTGACTTTACGGATGGTTCAATCCTCCGCGCGTATGACCTTAACGTCGATCAGATTCAAACGATGCACGTAGCGGAAGAGGCCCGTGACCTCACTGCGGATACTATCGGTGTCAATAACGATGGTCACTTGGATGCTCGTGGTCGTCGAATTGTGAACCTAGCAAACGCCGTGGATGACCGCGATGCTGTTCCGTTTGGTCAACTAAAGACCATGGACCAGAACTCATGGCAAGCAc                                                                                                                                                               <  1:130642/301‑1 (MQ=255)`  
                                                                                                                                                 `gCCGATGGCTACACGACCATCGAGTTACGTCGAGTAACCTCCACTACCGACCGATTGGTTGACTTTACGGATGGTTAAATCCTCCGCGCGTATGACCTTAACGTCGCTCAGATTCAAACGATGCACGTAGCGGAAGAGGCCCGTGACCTCACTGCGGATACTATCGGTGTCAATAACGATGGTCACTTGGATGCTCGTGGTCGTCGAATTGTGAACCTAGCGAACGCCGTGGATGACCGCGATGCTGTTCCGTTTGGTCAACTAAAGACCATGGACCAGAACTCATGGCAAGCAC‑GTAATg                                                                                                                                                        <  1:74313/301‑1 (MQ=255)`  
                                                                                                                                                     `aTGGCTACACGACCATCGAGTTACGTCGAGTAACCTCCACTACCGACCGATTGGTTGACTTTACGGATGGTTCAATCCTCCGCGCGTATGACCTTAACGTCGCTCAGATTCAAACGATGCACGTAGCGGAAGAGGCCCGTGACCTCACTGCGGATACTATCGGTGTCAATAACGATGGTCACTTGGATGCTCGTGGTCGT‑GAATTGTGAACCTAGCGAACGCCGTGGATGACCGCGATGCTGTTCCGTTTGGTCAACTAAAGACCATGGACCAGAACTCATGGCAAGCAC‑GTAATGAAGcc                                                                                                                                                   >  1:507018/1‑301 (MQ=255)`  
                                                                                                                                                     `aTGGCTACACGAACATCGAGTTACGTCGAGTAACCTCCACTACCGACCGATTGGTTGACTTTACGGATGGTTCAATCCTCCGCGCGTATGACCTTAACGTCGCTCAGATTCAAACGATGCACGTAGCGGAAGAGGCCCGTGACCTCACTGCGGATACTATCGGTGTCAATAACGATGGTCACTTGGATGCTCGTGGTCGTCGAATTGTGAACCTAGCGAACGCCGTGGATGACCGCGATGCTGTTCCGTTTGGTCAACTAAAGACCATGGACCAGCACTCATGGCAAGCAC‑GTAATGAAGc                                                                                                                                                    >  1:144333/1‑301 (MQ=255)`  
                                                                                                                                                      `cagCTACACGACCATCGAGTTACGTCGAGTAACCTCCACTACCGACCGATTGGTTGACTTTACGGATGGTTCAATCCTCCGCGCGTATGACCTTAACGTCGCTCAGATTCAAACGATGCACGTAGCGGAAGAGGCCCGTGACCTCACTGCGGATACTATCGGTGTCAATAACGATGGTCACTTGGATGCTCGTGGTCGTCGAATTGTGAACCTAGCGAACGCCGTGGATGACCGCGATGCTGTTCCGTTTGGTCAACTAAAGACCATGGACCAGAACTCATGGCAAGCAC‑GTAATGAAGcc                                                                                                                                                   <  1:306460/299‑1 (MQ=255)`  
                                                                                                                                                         `cTACACGACCATCGAGTTACGTCGAGTAACCTCCACTACCGACCGATTGGTTGACTTTACAGATGGTTCAATCCTCCGCGCGTATGACCTTAACGTCGCTCAGATTCAAACGATGCACGTAGCGGAAGAGGCCCGTGACCTCACTGCGGATACTATCGGTGTCAATAACGATGGTCACTTGGATGCTCGTGGTCGTCGAATTGTGAACCTAGCGCACGCCGTGGATGACCGGGTTCCTTTTCCTTTTGGCAAACTAAAGACCATGAACAGGAACTCATGG‑AAGGACGGTAATGAAGCCTTa                                                                                                                                                >  1:29771/1‑301 (MQ=255)`  
                                                                                                                                                            `aacGACCATCGAGTTACGTCGAGTAACCTCCACTACCGACCGATTGGTTGACTTTACGGATGGTTCAACCCTCCGCGCGTATGACCTTAACGTCGCTCAGATTCAAACGATGCACGTAGCGGAAGAGGCCCGTGACCTCACTGCGGATACTATCGGTGTCAATAACGATGGTCACTTGGATGCTCGTGGTCGTCGAATTGTGAACCTAGCGAACGCCGTGGATGACCGCGATGCTGTTCCGTTTGGTCAACTAAAGACCATGGACCAGAACTCATGGCAAGCAC‑GTAATGAAGCCTTACAg                                                                                                                                             <  1:717929/300‑1 (MQ=255)`  
                                                                                                                                                               `gACCATCGAGTTCCGTCGAGTAACCTCCACTACCGACCGATTGGTTGACTTTACGGATGGTTCAATCCTCCGCGCGTATGACCTTAACGTCGCTCAGATTCAAACGATGCACGTAGCGGAAGAGGCCCGTGACCTCACTGCGGATACTATCGGTGTCAATAACGATGGTCACTTTGATGCTCGTGGTCGTCGAATTGTGAACCTAGCGAACGCCGTGGATGACCGCGATGCTGTTCCGTTTGGTCAACTAAAGACCATGGACCAGAACTCATGGCAAGCAC‑GTAATGAAGCCTTACAGTTc                                                                                                                                          <  1:431833/301‑1 (MQ=255)`  
                                                                                                                                                                  `cATCGAGTTACGTCGAGTAACCTCCACTACCGACCGATTGGTTGACTTTACGGATGGTTCAATCCTCCGCGCGTATGACCTTAACGTCGCTCAGATTCAAACGATGCACGTAGCGGAAGAGGCCCGTGACCTCACTGCGGATACTATCGGTGTCAATAACGATGGTCACTTGGATGCTCGTGGTCGTCGAATTGTGAACCTAGCGAACGCCGTGGATGACCGCGATGCTGTTCCGTTTGGTCAACTAAAGACCATGGACCAGAACTCATGGCAAGCAC‑GTAATGAAGACTTACAGTTCCGt                                                                                                                                       <  1:69578/301‑1 (MQ=255)`  
                                                                                                                                                                    `tCGAGTTACGTCGAGTAACCTCCACTACCGACCGATTGGTTGACTTTACGGATGGTTCAATCCTCCGCGCGTATGACCTTAACGTCGCTCAGATTCAAACGATGCACGTAGCGGAAGAGGCCCGTGACCTCACTGCGGATACTATCGGTGTCAATAAAGATGGTCACTTGGATGCTCGTGGTCGTCGAATTGTGAACCTAGCGAACGCCGTGGATGACCGCGATGCTGTTCCGTTTGGTCAACTAAAGACCATGGACCAGAACTCATGGCAAGCAC‑GTAATGAAGCCTTACAGTTCCGTaa                                                                                                                                     <  1:734266/301‑1 (MQ=255)`  
                                                                                                                                                                    `tCGAGTTACGTCGAGTAACCTCCACTACCGACCGATTGGTTGACTTTACGGATGGTTAAATCCTCCGCGCGTATGACCTTAACGTCGCTCAGATTCAAACGATGCACGTAGCGGAAGAGGCCCGTGACCTCACTGCGGATACTATCGGTGTCAATAACGATGGTCACTTGGATGCTCGTGGTCGTCGAATTGTGAACCTAGCGAACGCCGTGGATGACCGCGATGCTGTTCCGTTTGGTCAACTAAAGACCATGGACCAGAACTCATGGCAAGCAC‑GTAATGAAGCCTTACAGTTCCGTaa                                                                                                                                     <  1:469086/301‑1 (MQ=255)`  
                                                                                                                                                                    `tCGAGTTACGGCGAGTAACCTCCACTACCGACCGATTGGTTGACTTTACGGATGGTTCAATCCTCCGCGCGTATGACATTAACGTCGCTCAGATTCAAACGATGCACGTAGCGGAAGAGGCCCGTGACCTCACTGCGGATACTATCGGTGTCAATAACGATGGTCACTTGGATGCTCGTGGTCGTCGAATTGTGAACCTAGCGAACGCCGTGGATGACCGCGATGCTGTTCCGTTTGGTCAACTAAAGACCATGGACAAGAACTCATGGCAAGCAC‑GTAATGAAGCCTTACAGTTCCGTaa                                                                                                                                     <  1:44552/301‑1 (MQ=255)`  
                                                                                                                                                                                 `aGTAACCTCCACTACCGACCGATTGGTTGACTTTACGGATGGTTCAATCCTCCGCGCGTATGACCTTAACGTCGCTCAGATTCAAACGATGCACGTAGCGGAAGAGGCCCGTGACCTCACTGCGGATACTATCGGTGTCAATAACGATGGTCACTTGGATGCTCGTGGTCGTCGAATTGTGAACCTAGCGAACGCCGTGGATGACCGCGATGCTGTTCCGTTTGGTCAACTAAAGACTATGGACCAGAACTCATGGCAAGCAC‑GTAATGCAGCCTTACAGTTCCGTAATGAGGCTGAGACt                                                                                                                        >  1:264342/1‑301 (MQ=255)`  
                                                                                                                                                                                      `ccTCCACTACCGACCGATTGGTTGACTTTACGGATGGTTCAATCCTCCGCGCGTATGACCTTAACGTCGCTCAGATTCAAACGATGCACGTAGCGGAAGAGGCCCGTGACCTCACTGCGGATACTATCGGTGTCAATAACGATGGTCACTTGGATGCTCGTGGTCGTCGAATTGTGAACCTAGCGAACGCCGTGGATGACCGCGATGCTGTTCCGTTTGGTCAACTAAAGACCATGGACCAGAACTCATGGCAAGCAC‑GTAATGAAGCCTTACAGTTCCGTAATGAGGCTGAGACTTTCAg                                                                                                                   >  1:64285/1‑301 (MQ=255)`  
                                                                                                                                                                                       `ctctACTACCGACCGATTGGTTGACTTTACGGATGGTTCAATCCTCCGCGCGTATGACCTTAACGTCGCTCATATTCAAACGATGCACGTAGCGGAAGAGGCCCGTGACCTCACTGCGGATACTATCGGTGTCAATAACGATGGTCACTTGGATGCTCGTGGTCGTCGAATTGTGAACCTAGCGAACGCCGTGGATGACCGCGATGCTGTTCCGTTTGGTCAACTAAAGACCATGGACCAGAACTCATGGCAAGCAC‑GTAATGAAGCCTTACAGTTCCGTAATGAGGCTGAGACTTTCAGa                                                                                                                  >  1:494087/1‑301 (MQ=255)`  
                                                                                                                                                                                       `cTCCACTACCGACCGATTGGTTGACTTTACGGATGGTTCAATCCTCCGCGCGTATGACCTTAACGTCGCTCAGATTCAAACGATGCACGTAGCGGAAGAGGCCCGTGACCTCACTGCGGATACTATCGGTGTCAATAACGATGGTCACTTGGATGCTCGTGGTCGTCGAATTGTGAACCTAGCGAACGCCGTGGATGACCGCGATGCTGTTCCGTTTGGTCAACTAAAGACCATGGACCAGAACTCATGGCAAGCAC‑GTAATGAAGCCTTACAGTTCCGTAATGAGGCTGAGACTTTCAGa                                                                                                                  >  1:237955/1‑301 (MQ=255)`  
                                                                                                                                                                                       `cTCCACTACCGACCGATTGGTTGACTTTACGGATGGTTCAATCCTCCGCGCGTATGACCTTAACGTCGCTCAGATTCAAACGATGCACGTAGCGGAAGAGGCCCGTGACCTCACTGCGGATACTATCGGTGTCAATAACGATGGTCACTTGGATGCTCGTGGTCGTCGAATTGTGAACCTAGCGAACGCCGTGGATGACCGCGATGCTGTTCCGTTTGGTCAACTAAAGACCATGGACCAGAACTCATGGCAAGCAC‑GTAATGAAGCCTTACAGTTCCGTAATGAGGCTGAGACTTTCAGa                                                                                                                  >  1:291721/1‑301 (MQ=255)`  
                                                                                                                                                                                         `ccACTACCGACCGATTGGTTGACTTTACGGATGGTTCAATCCTCCGCGCGTATGACCTTAACGTCGCTCAGATTCAAACGATGCACGTAGCGGAAGAGGCCCGTGACCTCACTGCGGATACTATCGGTGTCAATAACGATGGTCACTTGGATGCTCGTGGTCGTCGAATTGTGAACCTAGCGAACGCCGTGGATGACCGCGATGCTGTTCCGTTTGGTCAACTAAAGACCATGGACCAGAACTCATGGCAAGCAC‑GTAATGAAGCCTTACAGTTCCGTACTGAGGCTGAGACtctgtctct                                                                                                                >  1:461567/1‑293 (MQ=255)`  
                                                                                                                                                                                           `aCTACCGACCGATTGGTTGACTTTACGGATGGTTCAATCCTCCGCGCGTATGACCTTAACGTCGCTCAGATTCAACCGATGCACGTAGCGGAAGAGGCCCGTTACCTCACTGCGGATACTATCGGTGCCAATAACGATGGTCACTTGGATGCTCGTGGACGTCGAATTGTGAACCTAGCGAACGCCGTGGATGACCGCGATGATGTTTCGTTTGGTCAACTAAAGACCATGGACCAGAACTCATGGCAAGCAC‑GTAATGAAGCCTTTCAGTTCCGTAATGAGGCTTAGACTTTctcacacc                                                                                                              >  1:107676/1‑294 (MQ=255)`  
                                                                                                                                                                                                     `gaTTGGTTGACTTTACGGATGGTTCAATCCTCCGCGCGTATGACCTTAACGTCGCTCAGATTCAAACGATGCACGTAGCGGAAGAGGCCCGTAACCTCACTGCGGATACTATTGGTGTCAATAACGATGGTCACTTGGATGCTCGTGGGCGTCGAATTGTGAACCTAGCGAACGCCGTGGATGACCGCGATGCTGTTCCGTTTGGTCAACTAAAGACCATGGACCAGACCTCATGGCAAGAAC‑GACAAGAATCCTTACATTTCCGGAATGAGGCTGAAACTTTAAGAAAACAAGCGGCggg                                                                                                    >  1:387619/1‑301 (MQ=255)`  
                                                                                                                                                                                                            `tGACTTTACGGATGGTTCAATCCTCCGCGCGTATGACCTTAACGTCGCTCAGATTCAAACGATGCACGTAGCGGAAGAGGCCCGTGACCTCACTGCGGATACTATCGGTGTCAATAACGATGGTCACTTGGATGCTCGTGGTCGTCGAATTGTGAACCTAGCGAACGCCGTGGATGACCGCGATGCTGTTCCGTTTGGTCAACTAAAGACCATGGACCAGAACTCATGGCAAGCAC‑GTAATGAAGCCTTACAGTTCCGTAATGAGGCTGAGACTTTCAGAAACCAAGCGGAGGGCTTTAAg                                                                                             <  1:810153/301‑1 (MQ=255)`  
                                                                                                                                                                                                            `agACTTTACGGATGGTTCAATCCTCCGCGCGTATGACCTTAACGTCGCTCAGATTCAAACGATGCACGTAGCGGAAGAGGCCCGTGACCTCACTGCGGATACTATCGGTGTCAATAACGATGGTCACTTGGATGCTCGTGGTCGTCGAATTGTGAACCTAGCGAACGCCGTGGATGACCGCGATGCTGTTCCGTTTGGTCAACTAAAGACCATGGACCAGAACTCATGGCAAGCAC‑GTAATGAAGCCTTACAGTTCCGTAATGAGGCTGAGACTTTCAGAAACCAAGCGGAGGGATTTAAg                                                                                             <  1:63437/300‑1 (MQ=255)`  
                                                                                                                                                                                                              `aCTTTACGGATGGTTCAATCCTCCGCGCGTATGACCTTAACGTCGCTCAGATTCAAACGATGCACGTAGCGGAAGAGGCCCGTGACCTCACTGCGGATACTATCGGTGTCAATAACGATGGTCACTTGGATGCTCGTGGTCGTCGAATTGTGAACCTAGCGAACGCCGTGGATGACCGCGATGCTGTTCCGTTTGGTCAACTAAAGACCATGGACCAGAACTCATGGCAAGCAC‑GTAATGAAGCCTTACAGTTCCGTAATGAGGCTGAGACTTTCAGAAACCAAGCGGAGGGCTTTAAGaa                                                                                           <  1:726558/301‑1 (MQ=255)`  
                                                                                                                                                                                                                `ttttgggcttgTTCCATCCTCCGCGCGTATTACCTTAACGTTGCCCAGATTCAAACTATGCACGTAGCGGAAGAGACCCTTGACCTCAATGCGGATACTTTCGGTGTCAATAACGATGGTCACTTGGATGCTCGTGGTCGTCGAATTGTGAACCTAGCGAACGCCGTGGATGACCGCGATGCTGTTCCGTTTGGTCACCTAAAGACCATGGACCAGAACTCATGGCAAGCAC‑GTAATTAAGCCTTACAGTTCCGTAATGAGGCTGAGACTTTCAGAAACCAAGCGGAGGGCTTTAAGAACg                                                                                         <  1:550492/291‑1 (MQ=255)`  
                                                                                                                                                                                                                 `ttACGGATGGTTCAATCCTCCGCTCGTATGACCTTAACGTCGCTCAGATTCAAACGATGCACGTCGCGGAAGAGGCCCGTGACATCACCGCGGATACTATCGGTGTCAATAACGATGGTCACTTGGATGCTCGTGGTCGCCGAATTGTGAACCTAGCGAACGCCGTGGATGACAGCGATGCTGTTCCGTTTGGTCAACTAAAGACCATGGTCCAGAACTCATGGCAAGCAC‑GTACTGAAGCCTTACCGTTCCGTACTGAGGCTGAGACTTTCAGAACCCCAGCGGAGGGCTTTATGAACgc                                                                                        >  1:704782/1‑300 (MQ=255)`  
                                                                                                                                                                                                                     `ggATGGTTCAATCCTCCGCGCGTATGACCTTAACGTCGCTCAGATTCAAACGATGCACGTAGCGGAAGAGGCCCGTGACCTCACTGCGGATACTATCGGTGTCAATAACGATGGTCACTTGGATGCTCGTGGTCGTCGAATTGTGAACCTAGCGAACGCCGTGGATGACCGCGATGCTGTTCCGTTTGGTCAACTAAAGACCATGGACCAGAACTCATGGCAAGCAC‑GTAATGAAGCCTTACAGTTCCGTAATGAGGCTGAGACTTTCAGAAACCAAGCGGAGGGCCTTAAGAACGAGTcc                                                                                    >  1:776036/1‑301 (MQ=255)`  
                                                                                                                                                                                                                        `tGGTTCAATCCTCCGCGCGTATGACCTTAACGTCGCTCAGATTCAAACGATGCACGTAGCGGAAGAGGCCCGTGACCTCACTGCGGATACTATCGGTGTCAATAACGATGGTCACTTGGATGCTCGTGGCCGTCGAATTGTGAACCTAGCGAATGCCGTGGATGACCGGGATGCTGTTCCGTTTGGTCAACTAAATACCATGGACCAGAACTCATGGCAAGCAC‑GTAAGGAAGCCTTACAGGTCCGTAATGAGGCTGGGACTTTCAGAAACCAAGCGAAGGGCttgagaaacactgccagt                                                                                 >  1:611230/1‑285 (MQ=255)`  
                                                                                                                                                                                                                          `gTTCAATCCTCCGCGCGTATTACCTTAACGTCGCTCAGATTCAAACGATACACGTAGCGGAAGAGGCCCGTGACCGCACTGCGGATACTATCGGTGTCAATAACGATGGTCACTTGGATGCTCGTGGTCGTCGAATTGTGAACCTAGCGAACGCCGTGGATGACCGCGATGCTGTTCCGTTTGGTCAACTAAAGACCATGGACCAGAACTCATGGCAAGCAC‑GTAATGAAGCCTTACAGTTCCGTAATGAGGCTGAGACTTTCAGAAACCAAGCGGAGGGCTTTAAGAACGAGTCCAGTAc                                                                               <  1:315480/301‑1 (MQ=255)`  
                                                                                                                                                                                                                          `gTTCAATCCTCCGCGCGTATGACCTTAACGTCGCTCAGATTCAAACGATGCACGTAGCGGAAGAGGCCCGTGACCTCACTGCGGATACTATCGGTGTCAATAACGATGGTCACTTGGATGCTCGTGGTCGTCGAATTGTGAACCTAGCGAACGCCGTGGATGATCGCGATGCTGTTCCGTTTGGTCAACTAAAGACCATGGACCAGAACTCATGGCAAGCAC‑GTAATGAAGCCTTACAGTTCCGTAATGAGGCTGAGACTTTCAGAAACCAAGCGGAGGGCTTTAAGAACGAGTCCAGTAc                                                                               <  1:333960/301‑1 (MQ=255)`  
                                                                                                                                                                                                                          `gTTCAATCCTACGCGCGTATGACCTTAACGTCGCTCAGATTCAAACGATGCACGTAGCGGAAGAGGCCCGTGACCTCACTGCGGATACTATCGGTGTCAATAACGATGGTCACTTGGATGCTCGTGGTCGTCGAATTGTGAACCTAGCGAACGCCGTGGATGACCGCGATGCTGTTCCGTTTGGTCAACTAAAGACCATGGACCAGAACTCATGGCAAGCAC‑GTAATGAAGCCTTACAGTTCCGTAATGAGGCTGAGACTTTCAGGAACCCAGCGGAGGGCTTTAAGAACGAGTCCAGTAc                                                                               >  1:816856/1‑301 (MQ=255)`  
                                                                                                                                                                                                                             `cAATCCTCCGCGCGTATGACCTTAACGTCGCTCAGATTCAAACGATGCACGTAGCGGAAGAGGCCCGTGACCTCACTGCGGATACTATCGGTGTCAATAACGATGGTCACTTGGATGCTCGTGGTCGTCGAATTGTGAACCTAGCGAACGCCGTGGATGACCGCGATGCTGTTCCGTTTGGTCAACTAAAGACCATGGACCAGAACTCATGGCAAGCAC‑GTAATGAAGCCTTACAGTTCCGTAATGAGGCTGAGACTTTCAGAAACCAAGCGGAGGGCTTTAAGAACGAGTCCAGTACCaa                                                                            <  1:80323/301‑1 (MQ=255)`  
                                                                                                                                                                                                                               `aTCCTCCGCGCGTATGACCTTAACGTCGCTCAGATTCAAACGATGCACGTAGCGGAAGAGGCCCGTGACCTCACTGCGGATACTATCGGTGTCAATAACGATGGTCACTTGGATGCTCGTGGTCGTCGAATTGTGAACCTAGCGAACGCCGTGGATGACCGCGATGCTGTTCCGTTTGGTCAACTAAAGACCATGGACCAGAACTCATGGCAAGCAC‑GTAATGAAGCCTTACAGTTCCGTAATGAGGCTGAGACTTTCAGAAACCAAGCGGAGGGCTTTAAGAACGAGTCCAGTAcctgtt                                                                          >  1:619703/1‑297 (MQ=255)`  
                                                                                                                                                                                                                                    `ccGTGCGTATGACCTTAACGTCGCTCAGATTCAAACGATGCACGTAGCGGAAGAGGCCCGTGACCTCACTGCGGATACTATCGGTGTCAATAACGATGGTCACTTGGATGCTCTTGGTCGTCGAATTGTGAACCTAGCGAACGCCGTGGATGACCGCGATGCTGTTCCGTTTGGTCAACTAAAGACCATGGACCAGAACTCATGGCAAGCAC‑GTAATGAAGCCTTACAGTTCCGTAATGAGGCTGAGACTTTCAGAAACCAAGCGGAGGGCTTTAAGAACGAGTCCAGTACCAACGCTACg                                                                     >  1:4543/1‑301 (MQ=255)`  
                                                                                                                                                                                                                                       `cgcgTATGACCTTAACGTCGCTCAGATTCAAACGATGCACGTAGCGGAAGAGGCCCGTGACCTCACTGCGGATACTATCGGTGTCAATAACGATGGTCACTTGGATGCTCGTGGTCGTCGAATTGTGAACCTAGCGAACGCCGTGGATGACCGCGATGCTGTTCCGTTTGGTCAACTAAAGACCATGGACCAGAACTCATGGCAAGCAC‑GTAATGAAGCCTTACAGTTCCGTAATGAGGCTGAGACTTTCAGAAACCAAGCGGAGGGCTTTAAGAACGAGTCCAGTACCAACGCTACGAac                                                                  <  1:627890/301‑1 (MQ=255)`  
                                                                                                                                                                                                                                         `cggaTGACCTTAACGGCGCTCAGATTCAACCGATGCACGTAGCGTAAGAGGCCCGTGACCTCACTGCGGATACTATCGGTGTCAATAACGATGGGCACTTGGATGCTCGTGGTCGTCGAATTGTGAACCTAGCGAACGCCGTGGATGACCGCGATGCTGTTCCGTTTGGTAAACTAAAGACCATGGCCCAGAACTAATGGCAAGCAC‑GTAATGAAGCCTTACAGTTCCGTAATGAGGCTGAGACTTTCAGAAACCAAGCGGAGGGCTTTAAGAACGAGTCCAGTACCAACGCTACGAacac                                                                <  1:13716/298‑1 (MQ=255)`  
                                                                                                                                                                                                                                                `ccTTAACGTCGCTCAGATTCAAACGATGCACGTAGCGGAAGAGGCCCGTGACCTCACTGCGGATACTATCGGTGTCAATAACGATGGTCACTTGGATGCTCGTGGTCGTCGAATTGTGAACCTAGCGAACGCCGTGGATGACCGCGATGCTGTTCCGTTTGGTCAACTAAAGACCATGGACCAGAACTCATGGCAAGCAC‑GTAATGAAGCCTTACAGTTCCGTAATGAGGCTGAGACTTTCAGAAACCAAGCGGAGGGCTTTAAGAACGAGTCCAGTACCAACGCTACGAACACAAAGCAg                                                         >  1:54255/1‑301 (MQ=255)`  
                                                                                                                                                                                                                                                              `tcggagaggtgtataagagacaggAAGAGGCCCGTGACCTCACTGCGGATACTATCGGTGTCAATAACGATGGTCACTTGGATGCTCGTGGTCGTCGAATTGTGAACCTAGCGAACGCCGTGGATGACCGCGATGCTGTTCCGTTTGGTCAACTAAAGACCATGGACCAGAACTCATGGCAAGCAC‑GTAATGAAGCCTTACAGTTCCGTAATGAGGCTGAGACTTTCAGAAACCAAGCGGAGGGCTTTAAGAACGAGTCCAGTACCAACGCTACGAACACAAAGCAGTGGCGCGATGAGAc                                           <  1:580728/279‑1 (MQ=255)`  
                                                                                                                                                                                                                                                               `gATTCAAACGATGCACGTAGCGGAAGAGGCCCGTGACCTCACTGCGGATACTATCGGTGTCAATAACGATGGTCACTTGGATGCTCGTGGTCGTCGAATTGTGAACCTAGCGAACGCCGTGGATGACCGCGATGCTGTTCCGTTTGGTCAACTAAAGACCATTGACCAGAAATCATGGCAAGCAC‑GTAATGAAGCCTTACAGTTCCGTAATGAGGCTGAGACTTTCAGAAACCAAGCGGAGGGCTTTAAGAACGAGTCCAGTACCAACGCTACGAACACAAAGCAGTGGCGCGATGAGAcc                                          >  1:324386/1‑301 (MQ=255)`  
                                                                                                                                                                                                                                                                         `aTGCACGTAGCGGAAGAGGCCCGTGACCTCACTGCGGATACTATCGGTGTCAATAACGATGGTCACTTGGATGCTCGTGGTCGTCGAATTGTGAACCTAGCGAACGCCGTGGATGACCGCGATGCTGTTCCGTTTGGTCAACTAAAGACCATGGACCAGAACTCATGGCAAGCAC‑GTAATGAAGCCTTACAGTTCCGTAATGAGGCTGAGACTTTCAGAAACCAAGCGGAGGGCTTTAAGAACGAGTCCAGTACCAACGCTACGAACACAAAGCAGTGGCGCGATGAGACCAAGGGTTTct                                >  1:164730/1‑300 (MQ=255)`  
                                                                                                                                                                                                                                                                                               `gTGACCTCACTGCGGATACTATCGGTGTCAATAACGATGGTCACTTGGATGCTCGTGGTCGTCGAATTGTGAACCTAGCGAACGCCGTGGATGACCGCGATGCTGTTCCGTTTGGTCAACTAAAGACCATGGACCAGAACTCATGGCAAGCAC‑GTAATGAAGCCTTACAGTTCCGTAATGAGGCTGAGACTTTCAGAAACCAAGCGGAGGGCTTTAAGAACGAGTCCAGTACCAACGCTACGAACACAAAGCAGTGGCGCGATGAGACCAAGGGTTTCCGAGACGAAGCCAAGCGGTTCaa          >  1:201794/1‑301 (MQ=255)`  
                                                                                                                                                                                                                                                                                                `tGACCTCACTGCGGATACTATCGGTGTCAATAACGATGGTCACTTGGATGCTCGTGGTCGTCGAATTGTGAACCTAGCGAACGCCGTGGATGACCGCGATGCTGTTCCGTTTGGTCAACTAAAGACCATGGACCAGAACTCATGGCAAGCAC‑GTAATGAAGCCTTACAGTTCCGTAATTAGGCTGAGACTTTCAGAAACCAAGCGGAGGGCTTTAAGAACGAGTCCAGTACCAACGCTACGAACACAAAGCAGTGGCGCGATGAGACCAAGGGTTTCCGAGACGAAGCCAAGCGGTTCAAg         >  1:521602/1‑301 (MQ=255)`  
                                                                                                                                                                                                                                                                                                  `aCCTCACTGCGGATACTATCGGTGTCAATAACGATGGTCACTTGGATGCTCGTGGTCGTCGAATTGTGAACCTAGAGAACGCCGTGGATGACCGCGATGCTGTTCCGTTTGGTCAACTAAAGACCATGGACCAGAACTCATGGCAAGCAC‑GTAATGAAGCCTTACAGTTCCGTAATGAGGCTGAGACTTTCAGAAACCAAGCGGAGGGCTTTAAGAACGAGTCCAGTACCAACGCTACGAACACAAAGCAGTGGCGCGATGAGACCAAGGGTTTCCGAGACGAAGCCAAGCGGTTCAAGaa       <  1:250861/301‑1 (MQ=255)`  
                                                                                                                                                                                                                                                                                                   `ccTCACTGCGGATACTATCGGTGTCAATAACGATGGTCACTTGGATGCTCGTGGTCGTCGAATTGTGAACCTAGCGAACGCCGTGGATGACCGCGATGCTGTTCCGTTTGGTCAACTAAAGACCATGGACCAGAACTCATGGCAAGCAC‑GTAATGAAGCCTTACAGTTCCGTCATGAGGCTGAGACTTTCAGAAACCAAGCGGAGGGCTTTAAGAACGAGTCCAGTACCAACGCTACGAACACAAAGCAGTGGCGCCATGAGACCAAGGGGTTCCGAGACGAAGCCGAGCGGCTCACGAAt      >  1:660588/1‑301 (MQ=255)`  
                                                                                                                                                                                                                                                                                                   `ccTCACTGCGGATACTATCGGTGTCAATAACGATGGTCACTTGGATGCTCGTGGTCGTCGAATTGTGAACCTAGCGAACGCCGTGGATGACCGCGATGCTGTTCCGTTTGGTCAACTAAAGACCATGGACCAGAACTCATGGCAAGCAC‑GTAATGAAGCCTTACAGTTCCGTAATGAGGCTGAGACTTTCAGAAACCAAGCGGAGGGCTTTAAGAACGAGTCCAGTACCAACGCTACGAACACAAAGCAGTGGCGCGATGAGACCAAGGGTTTCCGAGACGAAGCCAAGCGGTTCAAGaaa      >  1:824385/1‑300 (MQ=255)`  
                                                                                                                                                                                                                                                                                                       `aCTGCGGATACTATCGGTGTCAATAACGATGGTCACTTGGATGCTCGTGGTCGTCGAATTGTGAACCTAGCGAACGCCGTGGATGACCGCGATGCTGTTCCGTTTGGTCAACTAAAGACCATGGACCAGAACTCATGGCAAGCAC‑GTAATGAAGCCTTACAGTTCCGTAATGAGGCTGAGACTTTCAGAAACCAAGCGGAGGGCTTTAAGAACGAGTCCAGTACCAACGCTACGAACACAAAGCAGTGGCGCGATGAGACCAAGGGTTTCCGAGACGAAGCCAAGCGGTTCAAGAATACgg  <  1:438599/301‑1 (MQ=255)`  

                                                                                                                                                                                                                                                                                                          `|`                                                                                                                                                                                                                                                                                                            
`TGATTTTAATATCCCGTTTGAGTATCTAGCCCGTAAGTTCGTAGTGGTAACTCTTATTGGTGTAGACCGAAAGGTCCTTACGATTAATACAGACTATCGCTTTGCTACACGTACTACTATCTCTCTGACAAAGGCTTGGGGTCCAGCCGATGGCTACACGACCATCGAGTTACGTCGAGTAACCTCCACTACCGACCGATTGGTTGACTTTACGGATGGTTCAATCCTCCGCGCGTATGACCTTAACGTCGCTCAGATTCAAACGATGCACGTAGCGGAAGAGGCCCGTGACCTCACTACGGATACTATCGGTGTCAATAACGATGGTCACTTGGATGCTCGTGGTCGTCGAATTGTGAACCTAGCGAACGCCGTGGATGACCGCGATGCTGTTCCGTTTGGTCAACTAAAGACCATGAACCAGAACTCATGGCAAGCAC‑GTAATGAAGCCTTACAGTTCCGTAATGAGGCTGAGACTTTCAGAAACCAAGCGGAGGGCTTTAAGAACGAGTCCAGTACCAACGCTACGAACACAAAGCAGTGGCGCGATGAGACCAAGGGTTTCCGAGACGAAGCCAAGCGGTTCAAGAATACGG  >  NC_001604.1/34677‑35272`

  

Alignment Legend

Aligned base mismatch/match (shaded by quality score): `ATCG/ATCG` `< 3 ≤` `ATCG/ATCG` `< 11 ≤` `ATCG/ATCG` `< 24 ≤` `ATCG/ATCG` `< 36 ≤` `ATCG/ATCG` `< 38 ≤` `ATCG/ATCG`

Unaligned base: `atcg`    Masked matching base: `atcg`    Alignment gap: `‑`    Deleted base: `‑`
